# Natural Selection, Not Mutation: Recombination in *Drosophila* Increases Diversity

**DOI:** 10.1371/journal.pbio.1001423

**Published:** 2012-11-13

**Authors:** Robin Mejia

**Affiliations:** Freelance Science Writer, Albany, California, United States of America

**Figure:**
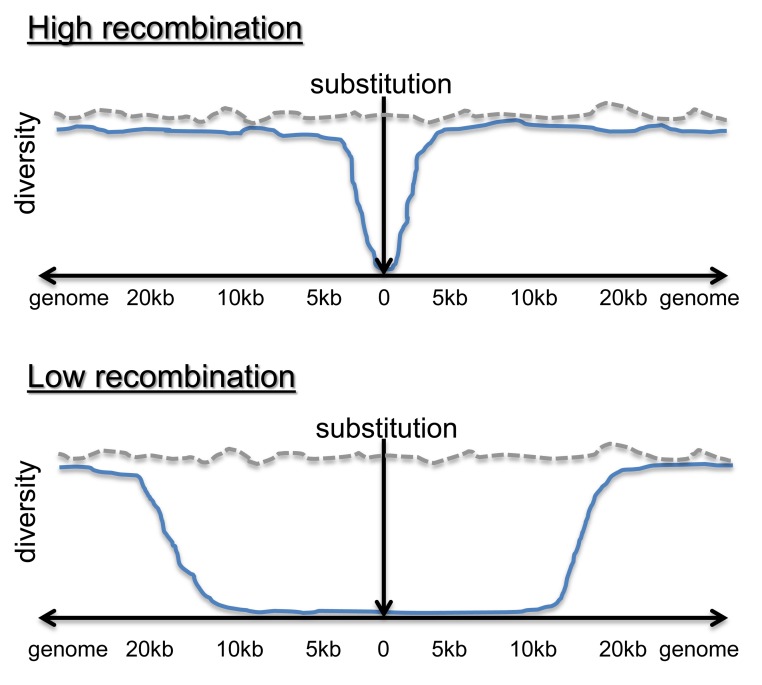
Local recombination rate is expected to affect baseline diversity (grey dotted line). The length of footprints in diversity from selection (blue line) depends on local recombination rate. Evidence from this study supports this simplified model.

Plants do it. Animals do it. People do it, too. During meiosis—the cell division that creates eggs and sperm—all these organisms mix the genetic material inherited from their mother and father. This mixing is called recombination, and it results from crossover events, where pairs of chromosomes swap genetic material. Evolutionary biologists have long known that recombination seems to increase genetic diversity. That is, high rates of recombination in a species are correlated with high rates of genetic diversity between individuals in that species.

What has been less clear is *how* recombination leads to increased diversity. Do crossover events predominantly cause mutations, or is their primary effect to break up linkage between genes? Genes that are linked—near each other on the same chromosome—are more likely to be inherited together. By breaking up linkage, recombination makes it easier for natural selection to target individual genes while avoiding the potentially disadvantageous effect of simultaneously reducing diversity at neighboring genes (a phenomenon known as “Hill-Robertson interference”).

To assess whether recombination causes mutations, researchers have previously looked at recombination rates at certain points on the same chromosome of closely related species and assessed whether there was increased genetic divergence—genetic variation *between* species—at those points. They found no increase in divergence, leading to the conclusion that the association between diversity and recombination was not predominantly caused by mutations. However, these analyses suffered from the implicit assumption that recombination rates were the same in related species. Recently, a number of studies have found that this is not always the case; even in closely related species, local recombination rates can vary greatly, raising questions about work that has not taken this variability into account and leaving room for the mutation hypothesis to resurface.

In this issue of *PLOS Biology*, Suzanne McGaugh, Mohamed Noor, and colleagues at Duke University and the University of Wisconsin-Madison settle the question, at least for fruit flies. Using relatives of the well-known *Drosophila melanogaster*, the researchers compared the local genetic variation within and between species (i.e., diversity and divergence, respectively) at regions of the flies' chromosomes that they knew to have similar recombination rates.

In both *D. pseudoobscura* and *D. miranda*, local recombination rates vary throughout the genome. For this experiment, McGaugh and colleagues created fine-scale maps of recombination rates for both species, covering 43 percent of the *D. pseudoobscura* genome, and 31 percent of *D. miranda*. Using these maps, they located regions of the genomes that showed similar rates of recombination over several generations. To determine whether recombination was associated with genetic divergence between the two species, as would be expected if mutation were playing a role in increasing genetic diversity near recombination sites, they modeled the relationship between recombination and divergence. They also modeled the relationship between local recombination in *D. pseudoobscura* and local increases in genetic diversity (within-species variation).

The team's findings were unambiguous: the rate of recombination in a region had no relationship to the genetic *divergence*. However, within the *D. pseudoobscura* genome, the local rate of recombination was correlated with overall genetic *diversity*. They also documented local effects that appear to be due to natural selection: in the immediate vicinity of non-synonymous substitutions—mutations that alter the amino acid sequence of a protein—they documented “hitchhiking” of non-coding genetic diversity, which would be expected if evolutionary selective sweeps had fixed the primary adaptive mutation.

In addition, the team made ultrafine-scale maps of a subset of the regions covered by the fine-scale maps. These provided a more detailed view of recombination rates in the two species than had been seen before. Using the ultrafine-scale maps, the researchers saw even greater local variations in recombination rates than showed up on the fine-scale maps. For example, in one region, where the fine-scale map had shown a range of 4.4–5.6 cM/Mb (centimorgans per megabase), the ultrafine-scale version revealed a much wider range of 3.5–21.1 cM/Mb. They conclude that recombination rates in different regions of a genome may vary considerably more than previously realized, and that recombination rates measured using fine-scale maps are likely crude averages of more wildly varying rates over even smaller regions.

The researchers note that there is much left to learn about how recombination rates vary at extremely fine scales within species, and also how recombination patterns vary between species and taxa. They note that further study is needed into the mechanisms that determine the distribution of recombination events, especially as *Drosophila* lack several proteins normally considered crucial to meiotic crossover and DNA double-strand break repair in other organisms.

**McGaugh SE, Heil CSS, Manzano-Winkler B, Loewe L, Goldstein S, et al. (2012) Recombination Modulates How Selection Affects Linked Sites in** ***Drosophila*** **. doi:10.1371/journal.pbio.1001422**

